# Severe pneumonia due to Nocardia otitidiscaviarum identified by mass spectroscopy in a cotton farmer: A case report and literature review: Erratum

**DOI:** 10.1097/MD.0000000000007646

**Published:** 2017-07-21

In the article, “Severe pneumonia due to Nocardia otitidiscaviarum identified by mass spectroscopy in a cotton farmer: A case report and literature review”,^[1]^ which appeared in Volume 96, Issue 13 of *Medicine*, Chen Liu, Mei Feng, and Jin Zhu should all have been listed as contributing equally.

## References

[1] Liu C Feng M Zhu J Severe pneumonia due to Nocardia otitidiscaviarum identified by mass spectroscopy in a cotton farmer: A case report and literature review. *Medicine.* 2017 96:e6526. 28353613 10.1097/MD.0000000000006526 PMC5380297

